# Comparative Chiral Separation of Thalidomide Class of Drugs Using Polysaccharide-Type Stationary Phases with Emphasis on Elution Order and Hysteresis in Polar Organic Mode

**DOI:** 10.3390/molecules27010111

**Published:** 2021-12-24

**Authors:** Mohammadhassan Foroughbakhshfasaei, Máté Dobó, Francisc Boda, Zoltán-István Szabó, Gergő Tóth

**Affiliations:** 1 Department of Pharmaceutical Chemistry, Semmelweis University, Hőgyes E. Str. 9, H-1085 Budapest, Hungary; ardal.okok@gmail.com (M.F.); dobomate99@gmail.com (M.D.); 2 Department of General and Inorganic Chemistry, George Emil Palade University of Medicine, Pharmacy, Science, and Technology of Targu Mures, Gh. Marinescu 38, RO-540139 Targu Mures, Romania; francisc.boda@umfst.ro; 3 Department of Pharmaceutical Industry and Management, George Emil Palade University of Medicine, Pharmacy, Science, and Technology of Targu Mures, Gh. Marinescu 38, RO-540139 Targu Mures, Romania; zoltan.szabo@umfst.ro

**Keywords:** chiral HPLC, enantiomer elution order, enantioseparation, hysteresis, polysaccharide-type stationary phase, polar organic mode, thalidomide

## Abstract

The enantioseparation of four phthalimide derivatives (thalidomide, pomalidomide, lenalidomide and apremilast) was investigated on five different polysaccharide-type stationary phases (Chiralpak AD, Chiralpak AS, Lux Amylose-2, Chiralcel OD and Chiralcel OJ-H) using neat methanol (MeOH), ethanol (EtOH), 1-propanol (PROP), 2-propanol (IPA) and acetonitrile (ACN) as polar organic mobile phases and also in combination. Along with the separation capacity of the applied systems, our study also focuses on the elution sequences, the effect of mobile phase mixtures and the hysteresis of retention and selectivity. Although on several cases extremely high resolutions (*R*_s_ > 10) were observed for certain compounds, among the tested conditions only Chiralcel OJ-H column with MeOH was successful for baseline-separation of all investigated drugs. Chiral selector- and mobile-phase-dependent reversals of elution order were observed. Reversal of elution order and hysteresis of retention and enantioselectivity were further investigated using different eluent mixtures on Chiralpak AD, Chiralcel OD and Lux Amylose-2 column. In an IPA/MeOH mixture, enantiomer elution-order reversal was observed depending on the eluent composition. Furthermore, in eluent mixtures, enantioselectivity depends on the direction from which the composition of the eluent is approached, regardless of the eluent pair used on amylose-based columns. Using a mixture of polar alcohols not only the selectivities but the enantiomer elution order can also be fine-tuned on Chiralpak AD column, which opens up the possibility of a new type of chiral screening strategy.

## 1. Introduction

Enantioseparations are generally more time-consuming to develop compared to achiral separations. Understanding enantioseparation at the molecular level is a basic requirement to achieve a rational approach to chiral method development. Therefore, in-depth investigation of enantioseparation mechanisms is crucial to increase predictability. Among the several types of chiral selectors available on the market, polysaccharide-type chiral stationary phases (CSPs) are probably the most widely applied for chiral analysis in HPLC and in supercritical fluid chromatography (SFC) [1,2,3,4,5], which makes it critical to investigate their enantiorecognition properties even further. The versatile applicability of these CSPs in chiral HPLC was demonstrated in normal phase (hydrocarbon-alcohol mobile phases) [6], reversed phase (aqueous-organic mobile phases) [7], but also in polar organic mode (PO) [8,9]. In PO polar organic solvents, neat alcohols (mainly methanol (MeOH), ethanol (EtOH) and 2-propanol (IPA)) or acetonitrile (ACN) or their combinations are used as the mobile phase. This separation mode exhibits several advantages: versatility due to the different nature of applied solvents as mobile phases, shorter run time, high efficiency and high solubility of analyte in the mobile phase [8,9,10,11,12]. The elution order is crucial in enantiomeric purity analysis. Impurities eluting close to the main peak, can be quantified with better accuracy and precision when they elute before the major peak. In recent years, there were numerous studies regarding enantiomeric elution-order (EEO) reversals in PO mode using polysaccharide-type CSPs reported based on the structure of chiral selector, mobile phase constituents, type and concentration of acidic or basic additives, as well as column temperature [13,14,15,16,17,18,19,20]. In a recent article, Horváth et al. demonstrated that different mixtures of MeOH and IPA as a mobile phase can alter the structure of the amylose tris(3,5-dimethylphenylcarbamate) chiral selector, resulting in EEO reversals, depending on the IPA/MeOH proportions. Besides EEO reversal, a very interesting phenomenon called hysteresis was also observed on columns containing the abovementioned chiral selector: enantioselectivities and analyte retention times depended on the direction from which the composition (IPA or MeOH) of the eluent was approached [21]. This observation can be utilized as a novel screening method in PO mode [22].

**Figure 1:**
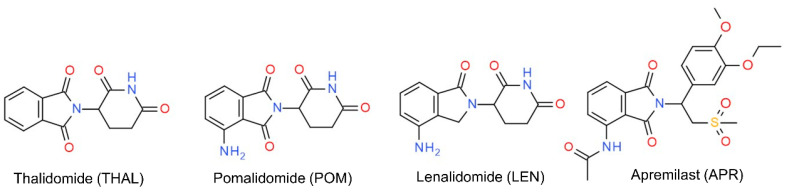
The structure of the investigated thalidomide derivatives.

After thalidomide (THAL) was reinstated in therapy, first as a treatment for erythema nodosum leprosum, later for multiple myeloma, the interest in thalidomide derivatives significantly increased [23]. This resulted in the approval of two structurally similar derivatives, namely pomalidomide (POM) and lenalidomide (LEN) as well as apremilast (APR), the structure of which shows a larger difference (Figure 1).

POM and LEN are drugs of hope in multiple myeloma, while APR is used for the treatment of psoriasis and psoriatic arthritis. In the case of the latter compound, the more potent *S*-enantiomer is marketed, which is possible because its structure lacks the acidic hydrogen at the chiral center and therefore has a higher stability against racemization [24]. In contrast the immunomodulatory drugs (IMIDs) (THAL, POM and LEN) are marketed as racemates, due to the fast in vivo racemization of these compounds; however, the *S*-enantiomers have been reported to be more potent. Several approaches are currently explored to stabilize the eutomers of IMIDs which underline even further the importance of the chirality of these drugs [25]. As a continuation of our recent studies regarding enantio-separation of THAL class of drugs [12,26,27,28,29], the aim of the present study was to investigate the enantiodiscriminating properties of a wide variety of polysaccharide-type CSPs with five solvents: ACN, MeOH, EtOH, IPA and propanol (PROP)) in PO mode. Along with the separation capacity of the applied systems, our study concentrates on the elution sequences in neat solvents and in different mixtures. Hysteresis in different polysaccharide-based columns was also examined.

## 2. Results

### 2.1. General Characterization of Enantioseparations

**Figure 2:**
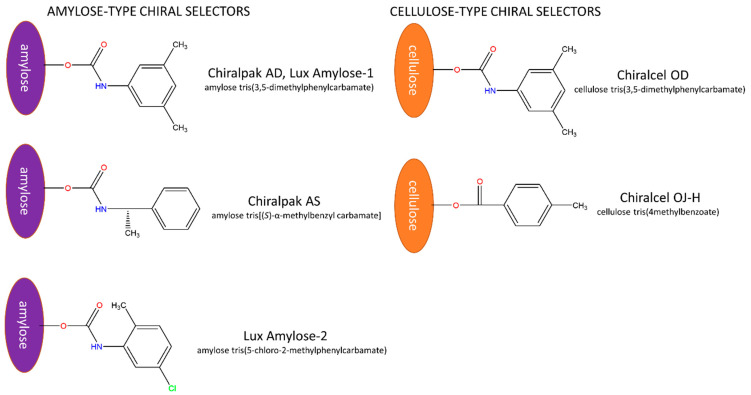
The structure of the chiral selectors.

A screening for the enantioseparation of the four drugs using five different polysaccharide-type CSPs, including three amylose-based columns, Chiralpak AD, Chiralpak AS and Lux Amylose-2 as well as two cellulose-based columns, Chiralcel OD and Chiralcel OJ-H have been performed in polar organic mode using neat MeOH, EtOH, PROP, IPA, and ACN as the mobile phase in a total of 25 separation systems. The chemical structures of the five chiral selectors are depicted in Figure 2.

**Figure 3:**
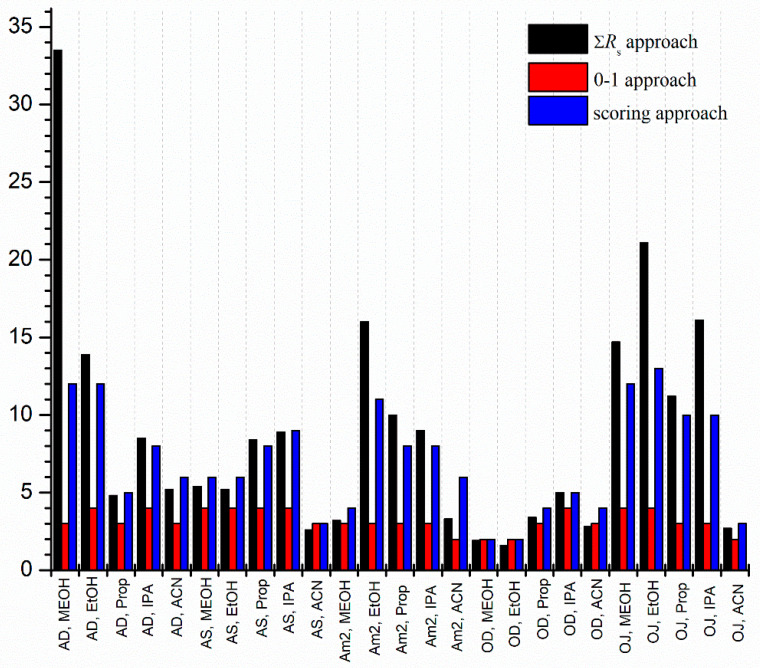
Characterization of the success of the applied separation system. Separation conditions were as indicated in Section 3.

Chromatographic parameters such as retention time of the first (*t*_1_) and second eluting enantiomer (*t*_2_), resolution (*R*_s_) and elution sequence are summarized in Table 1 for all the applied systems using constant 0.5 mL/min flow rate and 20 °C column temperature. Some representative chromatograms are depicted in Supplementary Figure S1. It can be seen that there are several cases where baseline separation can be achieved for the analytes and in many cases extremely high *R*_s_ (*R*_s_ > 10) values were also obtained. The results indicate that polysaccharide-type CSPs under polar organic conditions are highly versatile for the enantioseparation of THAL class of drugs. There are two approaches, often applied to compare the separation capacity of applied chromatographic systems. In the first one, the sum of the *R*_s_ values is calculated; however, in our case, this approach would distort comparison due to the extremely high resolutions obtained on the Chiralpak AD column. Moreover, it should be noted that high *R*_s_ values were accompanied with long analysis times, which is detrimental in practice. The other approach is to use 0 or 1 values depending on whether the separation is successful (enantiorecognition observed) or not. This approach, however, overestimates the separation with low resolutions. To characterize the enantioseparation capacity of our applied systems, a special scoring strategy was used. According to the *R*_s_ value of each individual run, a score from 0 to 4 was given, based on the performance of the separation system (*R*_s_ = 0 = 0, 0 < *R*_s_ < 1.5 = 1, 1.5 < *R*_s_ < 2.5 = 2, 2.5 < *R*_s_ < 4 = 3, 4 < *R*_s_ = 4) and the sum of score values for all four drugs were calculated in each applied separation systems. The comparison of enantioseparation systems with all three approaches is illustrated in Figure 3 and Supplementary Figure S2.

Based on the scoring strategy, the best results were achieved using Chiralcel OJ-H with EtOH and MeOH, Chiralpak AD with MeOH and EtOH, and Lux Amylose-2 with EtOH. It can also be seen that the sum *R*_s_ approach overestimates the Chiralpak AD column with MeOH because of the extremely high resolution observed for THAL and POM. The 0–1 approach is not helpful in this case due to the great number of successful enatio-separations, and the fact that this strategy would not differentiate between the applied separation systems. Results also indicated that polar, protic alcohols deliver the most promising results for the separation of these drugs (EtOH and MeOH). Despite of high score/*R*_s_ values, not all of the abovementioned conditions were successful in providing baseline separation for all four pairs of enantiomers. In many cases high *R*_s_ values were achieved for all of the IMIDs (in order THAL > POM > LEN) but not for APR. For example, on Chiralpak AD with MeOH or on Lux Amylose-2 with EtOH all of the IMIDs were baseline resolved, but no separation was observed for APR enantiomers. This observation can be explained by the structural differences between APR and the other IMIDs. Among all investigated separation systems, baseline separations for all enantiomeric pairs were achieved only by using Chiralcel OJ-H with MeOH.

Some interesting observations were revealed by comparing the separation systems with different alcohol type modifiers. Interestingly, much higher *R*_s_ values were achieved with EtOH in comparison to MeOH on Lux Amylose-2 column, which shows that EtOH can be an advantageous alternative mobile phase for MeOH. An additional example that shows the difference in using MeOH and EtOH as a neat mobile phase is that APR was not separated on Chiralpak AD using MeOH, but by changing the mobile phase to neat EtOH, an *R*_s_ of 1.2 can be achieved. Similar differences can be observed in the case of the PROP-IPA pair as well. For example, in the case of THAL using Chiralcel OD column, enantioseparation was not achieved using PROP, but using IPA, an *R*_s_ of 1.15 was obtained. However, when switching from MeOH or EtOH to PROP or IPA, the most striking differences in the enantiorecognition process are apparent by the reversal of elution order for THAL and POM on Chiralpak AD (see more details in Section 2.2 Enantiomer elution order).

Comparison of the retention factors of enantiomers revealed that the lowest retention was achieved for ACN in all cases, regardless of the CSPs employed. In the case of IMIDs, the low retention factors obtained with ACN were also accompanied by the lowest *R*_s_ values. However, for APR on the Chiralpak AD column, the highest *R*_s_ can be observed using ACN, which could be explained by the higher number of hydrogen bond acceptor moieties available in the APR structure. The order of *k*_1_ values for alcoholic type mobile phases usually increases in the order of MeOH < EtOH < PROP < IPA. An interesting exception can be observed on Chiralpak AD column, where *k*_1_ in IPA is significantly lower than that in MeOH or EtOH. This is most frequently explained by the fact that alcohols of different size and bulkiness can be incorporated in the CSPs structure and can also induce conformational changes in the helical structure of the polysaccharide selectors, resulting in different stereoenvironments [30].

### 2.2. Enantiomer Elution Order

EEO reversal in liquid chromatographic enantioseparations can be easily attained by using a chiral selector with the opposite stereochemical configuration. However, in the case of polysaccharide-type chiral selectors, which are available only in one single stereochemical configuration, this approach is not applicable. Observations regarding EEO, especially EEO reversals are crucial to better understand the recognition mechanism and to design novel chiral separation strategies [31,32,33,34]. Until now chiral selector- [4,6], mobile phase- [14,15], and temperature-dependent reversal of EEO [32,35] was observed in the literature.

In our study, chiral-selector and mobile-phase dependent reversal of EEO was observed in numerous cases, which are discussed in detail in next chapters. Moreover, it can be also noted that only small changes in the structure of analytes can often result in opposite EEO.

#### 2.2.1. The Effect of the Type of Chiral Selector

Reversal of EEO was observed for THAL and POM on Chiralpak AD using MeOH or EtOH in comparison to Chiralcel OD using the same neat mobile phases. These observations can most probably be explained based on the differences in the backbone (amylose vs. cellulose) of the chiral selector. Both polymeric chiral selectors present the same side chain (3,5-dimethylphenylcarbamate), and the same monomeric *D*-glucopyranose building blocks; however, the difference between amylose and cellulose is that in the former, the monomeric units present α(1,4) glycosidic bonds, while in the latter, these are *β*(1,4) linkages. Reversal of elution order observed between CSPs based on amylose tris(3,5-dimethylphenylcarbamate) and cellulose tris(3,5-dimethylphenylcarbamate) are often described and frequently explained by the conformational difference between the two CSPs [36]. It is also interesting to note that these types of EEO reversals in the case of POM and THAL were not observed if neat PROP or IPA was employed as the mobile phase.

Several examples for the reversal in EEO due to change in the nature of substituents of chiral selectors were also observed. For example, EEO of LEN changed the Chiralpak AD to the Chiralpak AS column, regardless of the mobile phase, and EEO of THAL and POM changed from Chiralpak AD to Lux Amylose-2 with MeOH and EtOH. In these cases, the backbone of the chiral selectors is the same, only the pendant groups differ, which results in different enantiorecognition environments. Two examples regarding chiral selector dependent EEO reversal was depicted in Supplementary Figures S3 and S4.

#### 2.2.2. Mobile Phase-Dependent Reversal of Elution Order

The next type of EEO reversal observed is the mobile phase-dependent reversal of elution order. Based on the experimental data, two main patterns were observed using neat mobile phases, which resulted in EEO reversals:

1. Change from alcohol type modifier to ACN
2. Change from MeOH or EtOH to PROP or IPA

The EEO of THAL was reversed on Chiralpak AD by using ACN instead of MeOH or EtOH. This can be tentatively explained based on the different hydrogen bonding capabilities of MeOH or EtOH in comparison to ACN and/or by the solvent dependent changes in the tridimensional structure of the chiral selector [20,37,38,39]. Opposite EEO was achieved for POM and THAL on Chiralpak AD using MeOH or EtOH vs. PROP or IPA. It has already been shown that alcohols of different size and bulkiness can be incorporated in the CSPs structure and can also induce conformational changes in the helical structure of the polysaccharide selectors, which result in different stereoenviroment of the so-called chiral grooves that are found in these chiral selector structures [20,38,39].

### 2.3. Enantioseparation of Mobile Phase Mixtures. Hysteresis of Retention and Selectivity

Use of mobile phase mixture instead of neat solvents could display several advantages. Finding the optimal mixture can increase selectivity, reduce retention time, and improve peak shape. Moreover, further investigation of retention profile and the mechanism of EEO reversal in mobile phase mixtures could help for better understanding of the enantiorecognition mechanism. Based on the scouting study using neat solvent, the following combination was applied:

1. Combination of MeOH/IPA applied on Chiralpak AD for THAL and POM
2. Combination of MeOH/PROP applied on Chiralpak AD for THAL
3. Combination of MeOH/ACN and EtOH/ACN applied on Chiralpak AD for THAL
4. Combination of MeOH/IPA applied on Chiralcel OD for POM
5. Combination of MeOH/IPA applied on Lux Amylose-2 for POM

**Figure 4:**
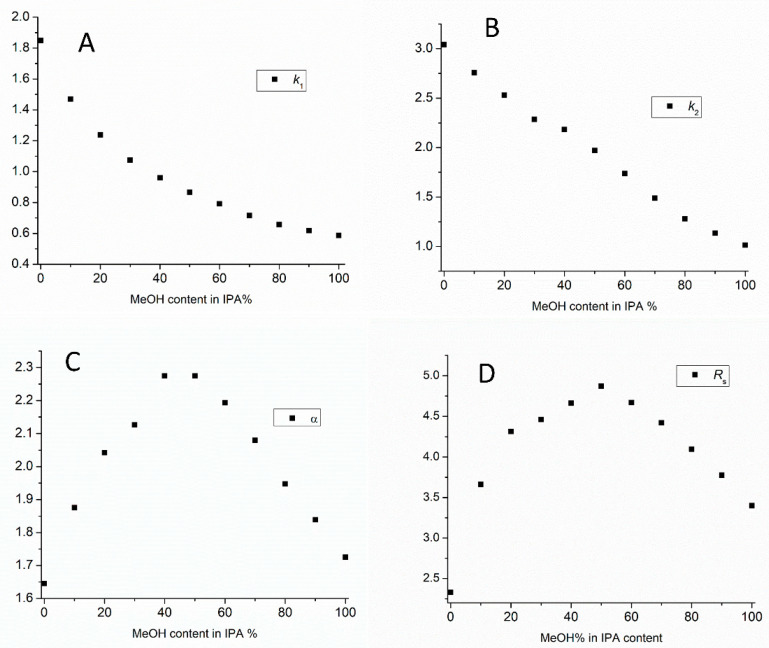
Plots of the retention factors ((**A**): *k*_1_, (**B**): *k*_2_), selectivity (**C**), and resolution factors (**D**) as a function of the MeOH content in IPA on Chiralcel OD column. (Chromatographic conditions: flow rate, 0.5 mL/min, column temperature 20 °C).

In all cases we started at 100% of the first eluent and added 10% of the second eluent in each step until we reached 100% of second eluent. The experiment was repeated in the opposite direction as well. On the Chiralpak AD column at a particular mobile phase composition, a co-elution was observed, but before this point and after this point the elution order was opposite, which indicates different enantiorecognition environments (Supplementary Figures S5–S7). The composition of solvent mixture used as eluent can provide several possible conformations of the chiral selector, which results in the different overall selectivity of the separation systems. Using mixtures of polar organic solvents affects not only enantioselectivity, but the EEO can also be fine-tuned. The investigation of the effect of eluent mixture on Chiralcel OD column is a nice example. In this case, using eluent mixtures instead of neat solvents can enhance enantioresolution, and can be a viable asset in chiral method development (Figure 4 and Supplementary Figure S8).

Figure 4 shows the effect of MeOH content in IPA on the retention, selectivity, and resolution of POM using Chiralcel OD column.

**Figure 5:**
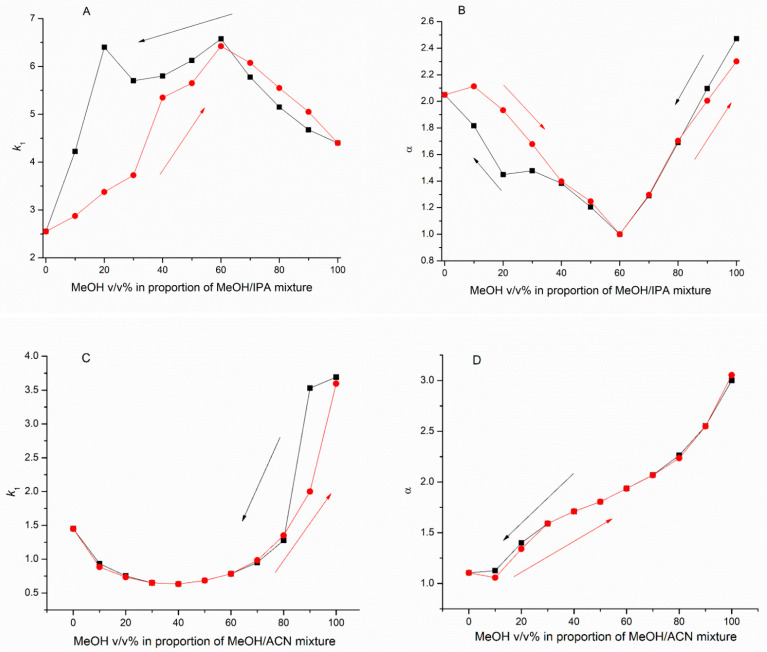
Some representative graphs of retention factor/separation factor vs. eluent composition (**A**): Retention factor of *S*-THAL in different MeOH/IPA compositions on Chiralpak AD column, (**B**): Separation factor of THAL enantiomers in different MeOH/IPA compositions on Chiralpak AD, (**C**): Retention factor of *S*-THAL in different MeOH/ACN compositions on Chiralpak AD column, (**D**): Separation factor of THAL enantiomers in different MeOH/ACN compositions on Chiralpak AD.

It can be seen that retention factors decreased as MeOH content increased. However, selectivity and resolution start to increase, up until 50% MeOH/IPA content. After this point, resolution decreased. This means that resolution and selectivity have a maximum at 50/50 MeOH/IPA content. Corresponding chromatograms are depicted in Figure 5. This finding can most probably be explained by various types of interactions that take place when using different polar organic mobile phase compositions and could offer interesting alternatives for method optimization.

Moreover, the analysis time or the peak shape can be easily fine-tuned by using eluent mixtures. In general, broad peak shapes obtained when using PROP or IPA can be overcome by adding ACN or MeOH to these eluents. These results further indicate the importance of using binary mobile phase compositions.

During our investigation regarding the different combination of mobile phases, it was observed that in many cases on the Chiralpak AD column using identical chromatographic conditions, substantially different results were observed in terms of retention factor and enantioselectivity. The results depended on the history of the eluents previously used on that particular column. It seems that the selectivity of the separation systems is highly influenced by the solvent that is used during the pre-conditioning of the CSP. This hysteretic behavior was described recently, and it was suggested that the history of eluents used previously on the column may alter the enantioselective environment of chiral selector by causing hindrance transition in the higher order structure of the CSP [21,22]. The hindrance transition effect in the structure of chiral selector can be explained by affecting the H-bond system through the application of different eluents. For example, different selectivity values and retention factors were achieved in several systems using Chiralpak AD column with totally identical composition of MeOH/IPA. The only difference in the case of these systems was that in one case the column was pre-conditioned using neat MeOH and in the other using neat IPA. Therefore, it can be assumed that in these systems the different results achieved are mainly affected by the change in the structure or stereonviroment of the chiral selector since the mobile phase is the same. Moreover, these history dependent differences were checked and also observed on Lux Amylose-1 column with the same chiral selector (amylose tris(3,5-dimethylphenylcarbamate) as the Chiralpak AD column. Our results confirm the recently reported observations by Horváth et al. [21] regarding the combination of MeOH/IPA as eluents, and further extend the possible mobile phase combinations displaying hysteretic behaviour to other mobile phase mixtures, such as MeOH/PROP, MeOH/ACN and EtOH/ACN. Thus, results indicate that the hysteretic behaviour on coated CSPs having amylose tris(3,5-dimethylphenylcarbamate) as chiral selectors is general, and it is not limited to the MeOH/IPA mixtures, but rather are more prominent in this system. Furthermore, the same investigations were also performed on Chiralcel OD and Lux Amylose-2 columns applying MeOH/IPA mixtures. Hysteretic behaviour could be observed on the amylose-based column, but not on the cellulose-based one (Supplementary Figure S9).

However, as the results show, the retention and selectivity differences are evident using the identical composition of mobile phases. These differences originate from the change in stereoenviroments of chiral selector, which is due to antecedents of the column. Representative graphs were plotted showing the concentration of one eluent in each binary system versus the retention factor of first enantiomer (*k*_1_), as well as selectivity value (α) (Figure 5).

**Figure 6:**
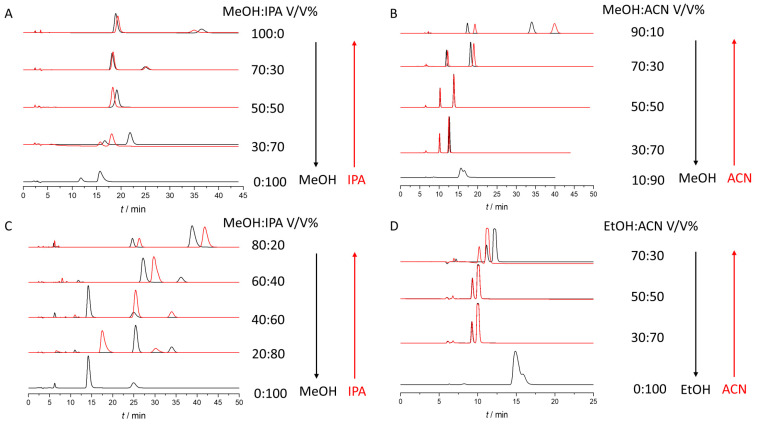
Chromatograms observed in different eluent compositions during the hysteresis study. (**A**): Enantioseparation of POM in different MeOH/IPA eluent mixtures using Chiralpak AD, (**B**): Enantioseparation of THAL in different MeOH/ACN eluent mixtures using Chiralpak AD, (**C**): Enantioseparation of THAL in different MeOH/IPA eluent mixtures using Chiralpak AD (**D**): Enantioseparation of THAL in different EtOH/ACN eluent mixtures using Chiralpak AD.

Representative chromatograms were depicted in Figure 6. The most pronounced hysteretic effect is visible in the case of THAL in the MeOH/IPA mixture on Chiralpak AD column (Figure 5) and POM in the MeOH/IPA mixture on Lux Amylose -2 column (Supplementary Figure S9), where the highest differences in retention factors were recorded. On the other hand, interestingly this hysteretic effect observed for THAL in the MeOH/PROP mixture is not as pronounced as in the MeOH/IPA mixture. Moreover, results also underline the observations from the preliminary experimental runs, that in chiral chromatography the substitution of IPA to PROP can lead to markedly different chromatographic behaviour of selected analytes. It is also observable that in the alcohol/ACN mixtures, the highest retention and selectivity are achieved by applying neat alcohol, which was not generally true when using the MeOH/IPA mixtures.

In general, by comparing the retention and selectivity profiles in different eluent mixtures, U-shaped curves are more common in the MeOH/PROP and EtOH/ACN mixture, displaying an increase in retention factor and selectivity when using high percentages of PROP or ACN, respectively. On the other hand, enantioseparation performance of the applied systems could be better at intermediate values of the MeOH/IPA mixtures. This behavior can be tentatively explained by a more pronounced change in the 3D structure of the chiral selector using a different composition of MeOH/IPA, which results in several transitional states of the chiral selector and affect its enantiomer recognition ability, or even cause the reversal in EEO. In the alcohol/ACN mixture it is assumed that the changes in enantiomeric recognition have more to do with the difference of the properties of solvents (e.g., hydrogen-bonding capacity) rather than pronounced changes in the 3D structure of selector itself.

## 3. Materials and Methods

### 3.1. Materials

Racemic THAL, LEN, POM, as well as *S*-POM, *R*- and *S*-APR were purchased from Beijing Mesochem Technology Co., Ltd. (Beijing, China). *R*-THAL and *S*-enantiomer-enriched LEN [29] were ordered from Sigma-Aldrich, Hungary (Budapest, Hungary). HPLC-grade MeOH, ethanol (EtOH), ACN, 1-propanol (PROP) and 2-propanol (IPA) were purchased from Merck (Darmstadt, Germany). Chiralpak AD (250 × 4.6 mm; particle size 10 µm) (based on amylose tris(3,5-dimethylphenylcarbamate)); Chiralcel OD (250 × 4.6 mm; particle size 10 µm) (based on cellulose tris(3,5-dimethylphenylcarbamate)), Chiralcel OJ-H (250 × 4.6 mm; particle size 5 µm) (based on cellulose tris(4methylbenzoate)) and Chiralpak AS (250 × 4.6 mm; particle size 10 µm) (based on amylose tris((S)-α-methylbenzyl carbamate)) were products of Daicel Corporation (Tokyo, Japan). Lux Amylose-1 (150 × 4.6 mm; particle size 5 µm) (based on amylose tris(3,5-dimethylphenylcarbamate)) and Lux Amylose-2 (150 × 4.6 mm; particle size 5 µm) (based on amylose tris(5-chloro-2-methylphenylcarbamate)) was ordered from Phenomenex (Torrance, CA, USA).

### 3.2. LC-UV Analysis

LC–UV analysis was carried out on an Agilent 1260 Infinity HPLC system (G1312B binary gradient pump, G1367E autosampler, G1315C diode array detector) (Agilent Technologies, Waldbronn, Germany). Agilent Masshunter B.04.00 software was used for instrument control and data analysis. HPLC separations were performed at 25 °C using a constant 0.5 mL/min flow rate. UV detection was performed at 210 nm. All stock solutions were prepared at 1 mg mL^−1^ in MeOH, and further dilutions were made with the same solvent. An injection volume of 1 μL was used and three parallel measurements were performed in each case. The enantiomer elution order (EEO) was determined by injecting enantiomers of known absolute configuration. For the determination of hold-up time, acetone was used. In the screening phase, neat alcohols (MeOH, EtOH, IPA or PROP) and ACN were used. Whenever an experiment required pre-treatment with either IPA, MeOH, EtOH or ACN, this was brought about by pumping 10 column volumes (CV) of the corresponding solvent through the column. Regarding hysteresis study we follow the protocol of the work of Németh and Simon [21]. In this work, the effect of different equilibration volumes (10 CV, 20 CV, 100 CV, 800 CV, 1600 CV and 2400 CV) on chromatographic behaviour were also tested. The chromatographic parameters (retention time, selectivity, resolution) were not affected by the applied equilibration volumes. In our cases the RSD% of the retention times for repetitive injections (*n* = 3) were below 0.3% in each case. During the investigation of hysteresis, we started our measurements with 100% MeOH (or EtOH) and we increased the amount of the other solvent by 10%, then we repeated the experiment in the opposite direction. Microcal Origin Pro 8 software (OriginLab, Northampton, MA, USA) was used for all linear regression analyses.

## 4. Conclusions

Successful enantioseparation of four phthalimide-analogue thalidomide derivatives was achieved on five different polysaccharide-type chiral selectors in a polar organic mobile phase mode using neat MeOH, EtOH, PrOP, IPA and can. In almost all cases, successful separations were achieved, especially in the case of IMIDs. On Chiralpak AD column, extremely high-resolution values were obtained in some cases; however, all the four enantiomeric pairs could be baseline-separated only on the Chiralcel OJ-H column. Comparing the effect of the applied mobile phase, it can be observed that MeOH delivered the most promising results, regardless of the applied stationary phase. Overall alcohol-type neat eluents were superior to ACN. Our study reports several cases of EEO reversal, depending on various characteristics of the chiral selectors and the composition of the mobile phase. Different backbone or substituents of the polysaccharide CSPs often lead to EEO reversal. The most interesting EEO reversals were observed on the Chiralpak AD column using different solvents in neat form or in mixture. Changing MeOH (EtOH) to ACN or MeOH (EtOH) to IPA (PROP) revealed cases of mobile phase dependent EEO reversals using neat eluents. By using eluent mixtures, however, better resolution and peak shape could be achieved, and moreover the EEO could be fine-tuned. On amylose tris(3,5-dimethylphenylcarbamate)- and amylose tris(5-chloro-2-methylphenylcarbamate)-containing CSPs, hysteresis of retention and selectivity were also observed in different eluent mixtures. The hysteresis phenomenon seems to be more general than an exceptional case for amylose-based columns. Our investigations reveal that the amylose-based chiral selectors most likely could exist in different stable conformational states and these stable states often display different enantiorecognition potentials. The measurements in these stable conformational states can pave the way to a novel chiral method development approach.

## Figures and Tables

**Table 1:** Chromatographic data, enantiomeric elution order (EEO), retention times of the enantiomers and resolution of the mobile phase and CSP screening for the chiral separation of thalidomide analogues in polar organic mode. Flow rate: 0.5 mL/min. Temperature: 20 °C.

		Thalidomide				Pomalidomide				Lenalidomide				Apremilast			
Column	Mobile Phase	*t*_1_	*t*_2_	*R*_s_	EEO	*t*_1_	*t*_2_	*R*_s_	EEO	*t*_1_	*t*_2_	*R*_s_	EEO	*t*_1_	*t*_2_	*R*_s_	EEO
Chiralpak AD	MeOH	29.8	76.4	14.4	S < R	19.8	37.2	12.8	S < R	4.9	5.5	6.3	S < R	26.1	-	-	-
	EtOH	49.8	64.2	4.4	S < R	28.2	39.0	5.1	S < R	22.8	26.9	3.2	S < R	22.7	25.0	1.2	R < S
	PROP	39.8	41.9	1.1	R < S	25.2	27.2	1.2	R < S	19.2	23.2	2.5	S < R	15.9	-	-	-
	IPA	14.7	25.5	4.8	R < S	14.4	16.3	1.9	R < S	10.9	12.8	1.4	S < R	16.2	17.1	0.8	R < S
	ACN	14.5	16.1	1.7	R < S	25.2	-	-	-	11	12.2	1.7	S < R	6.4	8.0	1.8	R < S
Chiralpak AS	MeOH	8.9	9.2	0.8	R < S	9.0	9.3	0.9	R < S	7.4	7.6	0.3	R < S	15.9	23.2	3.4	R < S
	EtOH	10.9	12.3	1.4	R < S	11.6	12.2	0.9	R < S	8.2	8.6	0.3	R < S	29.5	35.0	2.6	R < S
	PROP	17.7	19.4	1.2	R < S	17.0	19.3	1.5	R < S	10.7	11.7	1.1	R < S	28.1	42.1	4.6	R < S
	IPA	21.1	23.7	1.5	R < S	19.7	23.4	2.1	R < S	15.3	17.7	1.1	R < S	20.1	38.3	4.2	R < S
	ACN	6.4	6.7	0.6	R < S	6.9	7.3	0.8	R < S	5.5	5.8	1.2	R < S	6.6	-	-	-
Lux Amylose-2	MeOH	8.1	8.3	0.6	R < S	7.3	7.8	1.1	R < S	5.1	5.6	1.5	R < S	11.6	-	-	-
	EtOH	16.2	27.4	8.2	R < S	10.7	14.5	4.5	R < S	6.9	8.7	3.3	R < S	13.9	-	-	-
	PROP	18.9	25.8	5.3	R < S	12.8	16.5	3.4	R < S	8.4	9.6	1.3	R < S	21.2	-	-	-
	IPA	19.6	28.7	4.5	R < S	16.8	24.6	3.1	R < S	9.1	10.4	1.4	R < S	28.7	-	-	-
	ACN	5.3	5.6	1.6	R < S	4.3	5.2	1.7	R < S	5.3	-	-	-	4.28	-	-	-
Chiralcel OD	MeOH	11.4	11.8	0.8	R < S	9.7	12.3	3.4	R < S	9.3	-	-	-	17.3	-	-	-
	EtOH	18.9	19.6	0.8	R < S	10.7	11.6	0.8	R < S	10.4	-	-	-	24.9	-	-	-
	PROP	14.2	-	-	-	16.8	18.2	1.0	R < S	16.2	17.2	0.6	R < S	24.1	28.0	1.8	S < R
	IPA	17.3	18.7	1.2	R < S	17.4	24.7	2.3	R < S	18.1	22.1	0.9	R < S	52.6	61.4	1.2	S < R
	ACN	8.2	8.6	0.7	R < S	8.1	8.7	1.6	R < S	9.2	10.1	0.5	S < R	7.4	-	-	-
Chiralcel OJ-H	MeOH	11.1	14.3	7.2	R < S	13.1	14.5	2.9	R < S	7.6	8.0	3.1	R < S	41.2	46.0	1.5	S < R
	EtOH	16.0	26.6	10.7	R < S	17.2	22.7	5.5	R < S	10.3	13.2	4.3	R < S	55.0	59.3	0.6	S < R
	PROP	14.7	19.8	4.7	R < S	25.9	32.5	4.3	R < S	10.3	15.2	2.2	R < S	6.8	-	-	-
	IPA	20.7	36.7	9.7	R < S	20.2	26.5	3.9	R < S	11.6	16.0	2.5	R < S	51.5	-	-	-
	ACN	7.4	7.6	0.5	R < S	7.0	7.7	2.2	R < S	7.3	-	-	-	59.2	-	-	-

## Data Availability

The data presented in this study are available on request from the corresponding author.

## Supplementary Materials

The following are available online: Figure S1: Representative chromatograms, A: POM on Chiralpak AD, mobile phase: MeOH, B: POM on Chiralpak AD, mobile phase: IPA, C:THAL on Chiralpak AD, mobile phase: MeOH, D: LEN on Chiralcel OJ-H, mobile phase: PROP, E: THAL on Chiralcel OJ-H, mobile phase: EtOH, F:POM on Chiralcel OD, mobile phase: PROP, using constant 0.5 mL/min flow rate and 20 °C column temperature., Figure S2: Characterization of the success of the applied separation system in different approach (A): CSP, (B): mobile phase, (C): the separation success of the investigated molecules. Separation conditions were as indicated in Section 3, Figure S3: Example of backbone dependent enantiomer elution reversal–Pomalidomide on Chiralcel OD and Chiralpak AD column using 100% EtOH as mobile phase. Flow rate: 0.5 mL/min, temperature: 20 °C, Figure S4: Example of substituent dependent enantiomer elution reversal–Pomalidomide on Chiralpak AD and Lux amylose-2 column using 100% EtOH as mobile phase. Flow rate: 0.5 mL/min, temperature: 20 °C, Figure S5: Measurement of thalidomide in different MeOH/IPA mixtures on Chiralpak AD column. Flow rate: 0.5 mL/min. Temperature 25 °C, Figure S6: Measurement of pomalidomide in different MeOH/IPA mixtures on Chiralpak AD column. Flow rate: 0.5 mL/min. Temperature 25 °C, Figure S7: Measurement of thalidomide in different ACN/EtOH mixtures on Chiralpak AD column. Flow rate: 0.5 mL/min. Temperature 25 °C; Figure S8: POM on Chiralcel OD column using 100% MeOH (A), 50/50 MeOH/IPA mixtures (B), 100% IPA (C) as eluent. The Rs values are 3.40, 4.87 and 2.33, respectively, Figure S9: Representative graphs of retention factor/separation factor vs. eluent composition (A): Retention factor of R-POM in different MeOH/IPA compositions on Lux Amylose-2 column, (B): Separation factor of POM enantiomers in different MeOH/IPA compositions on Lux Amylose-2 column.
