# Supplementary material for: Comparative Chiral Separation of Thalidomide Class of Drugs Using Polysaccharide-Type Stationary Phases with Emphasis on Elution Order and Hysteresis in Polar Organic Mode
Source: Molecules. 2021 Dec 24;27(1):111. doi: 10.3390/molecules27010111 (PMC8746373; doi:10.3390/molecules27010111)
Supplement: Supplementary file 1 [file molecules-27-00111-s001.zip › molecules-1500240-supplementary.pdf]

Supplementary Information

Comparative chiral separation of thalidomide class of drugs using polysaccharide-type stationary phases with emphasis on elution order and hysteresis in polar organic mode

Mohammadhassan Foroughbakhshfasaei[1], Dobó Máté[1], Francisc Boda[2], Zoltán-István Szabó[3], Gergő Tóth[1*]

[1] Department of Pharmaceutical Chemistry, Semmelweis University, Hőgyes E. str. 9, Budapest H-1085, Hungary

[2] Department of General and Inorganic Chemistry, George Emil Palade University of Medicine, Pharmacy, Science, and Technology of Targu Mures, Gh. Marinescu 38, Targu Mures, RO-540139, Romania

[3] Department of Pharmaceutical Industry and Management, George Emil Palade University of Medicine, Pharmacy, Science, and Technology of Targu Mures, Gh. Marinescu 38, Targu Mures, RO-540139, Romania

***Corresponding author**: Gergő Tóth, Department of Pharmaceutical Chemistry, Semmelweis University, H-1092 Budapest, Hőgyes E. u. 9, Hungary.

Tel: +36 12170891; Fax: +36 12170891

Email address: toth.gergo@pharma.semmelweis-univ.hu;

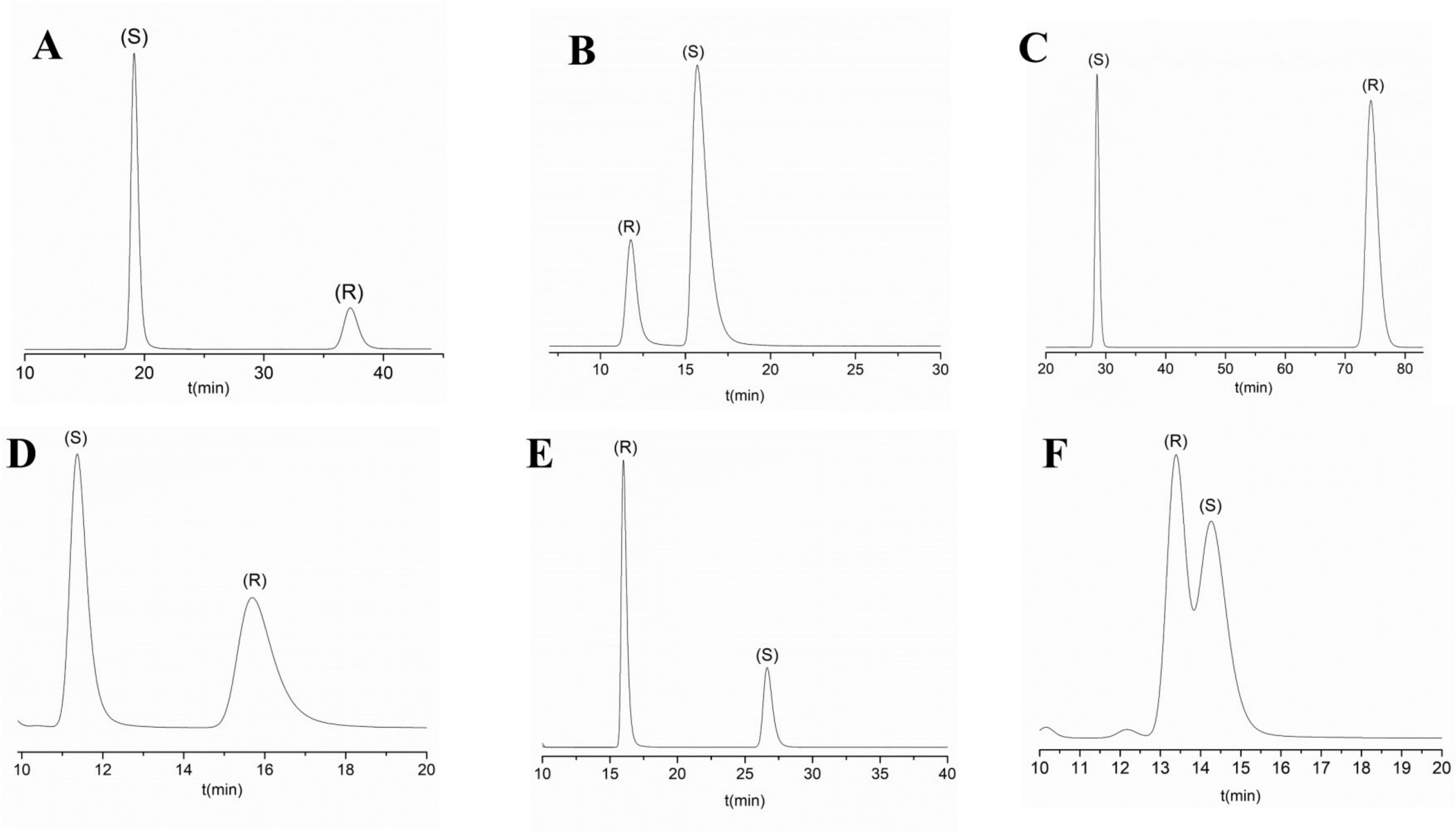

Supplementary Figure S1. Representative chromatograms, **A**: POM on Chiralpak AD, mobile phase: MeOH, **B**: POM on Chiralpak AD, mobile phase: IPA, **C**:THAL on Chiralpak AD, mobile phase: MeOH, **D**: LEN on Chiralcel OJ-H, mobile phase: PROP, **E**: THAL on Chiralcel OJ-H, mobile phase: EtOH, **F**:POM on Chiralcel OD, mobile phase: PROP, using constant 0.5 mL/min flow rate and 20 °C column temperature.

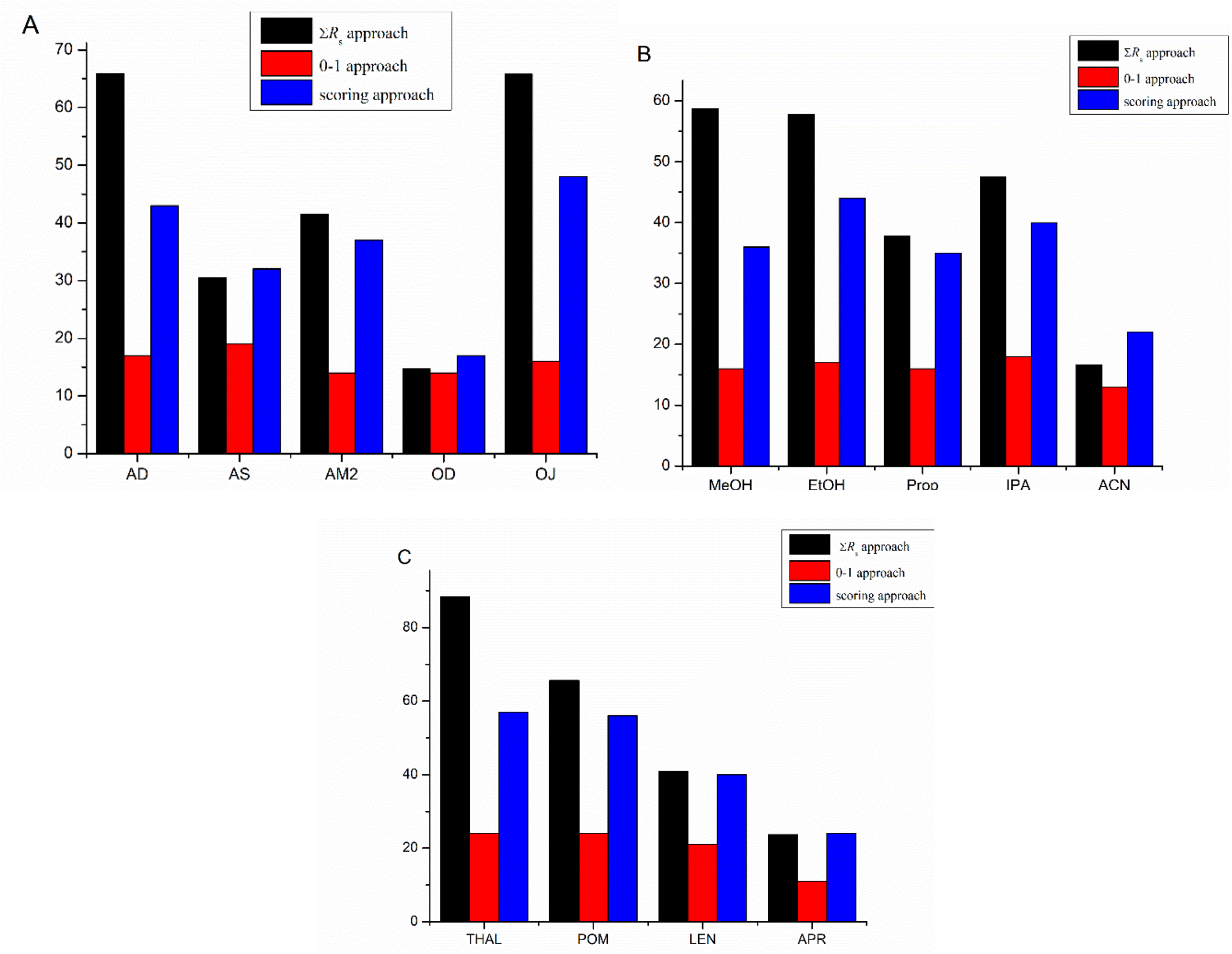

Supplementary Figure S2. Characterization of the success of the applied separation system in different approach A: CSP, B: mobile phase, C: the separation success of the investigated molecules. Separation conditions were as indicated in Section 3. (black: $\sum R_s$, red: 0-1 approach, blue: scoring approach)

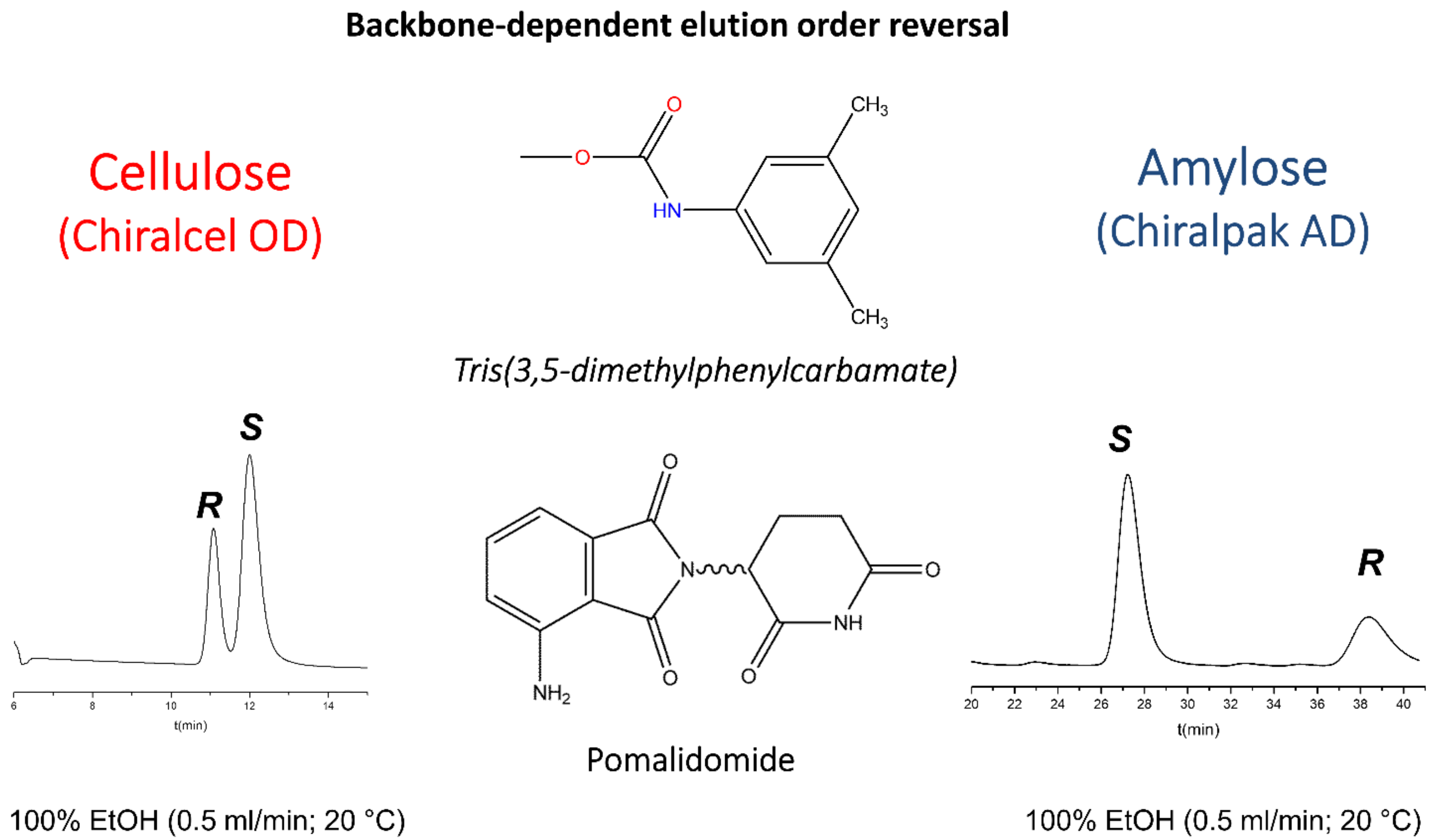

Supplementary Figure S3. Example of backbone dependent enantiomer elution reversal – POM on Chiralcel OD and Chiralpak AD column using 100% EtOH as mobile phase. Flow rate: 0.5 mL/min, temperature: 20 °C

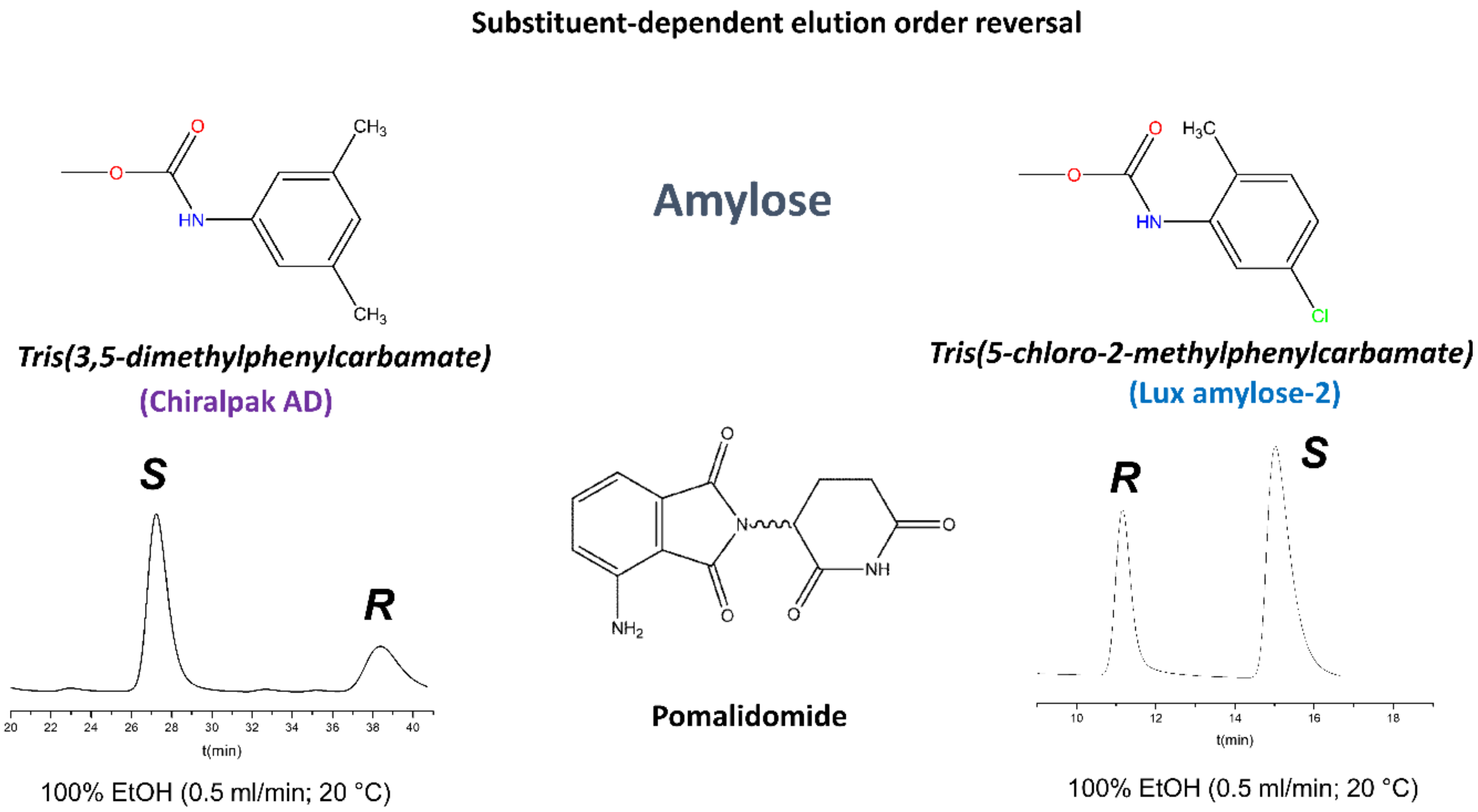

Supplementary Figure S4. Example of substituent dependent enantiomer elution reversal – POM on Chiralpak AD and Lux amylose-2 column using 100% EtOH as mobile phase. Flow rate: 0.5 mL/min, temperature: 20 °C

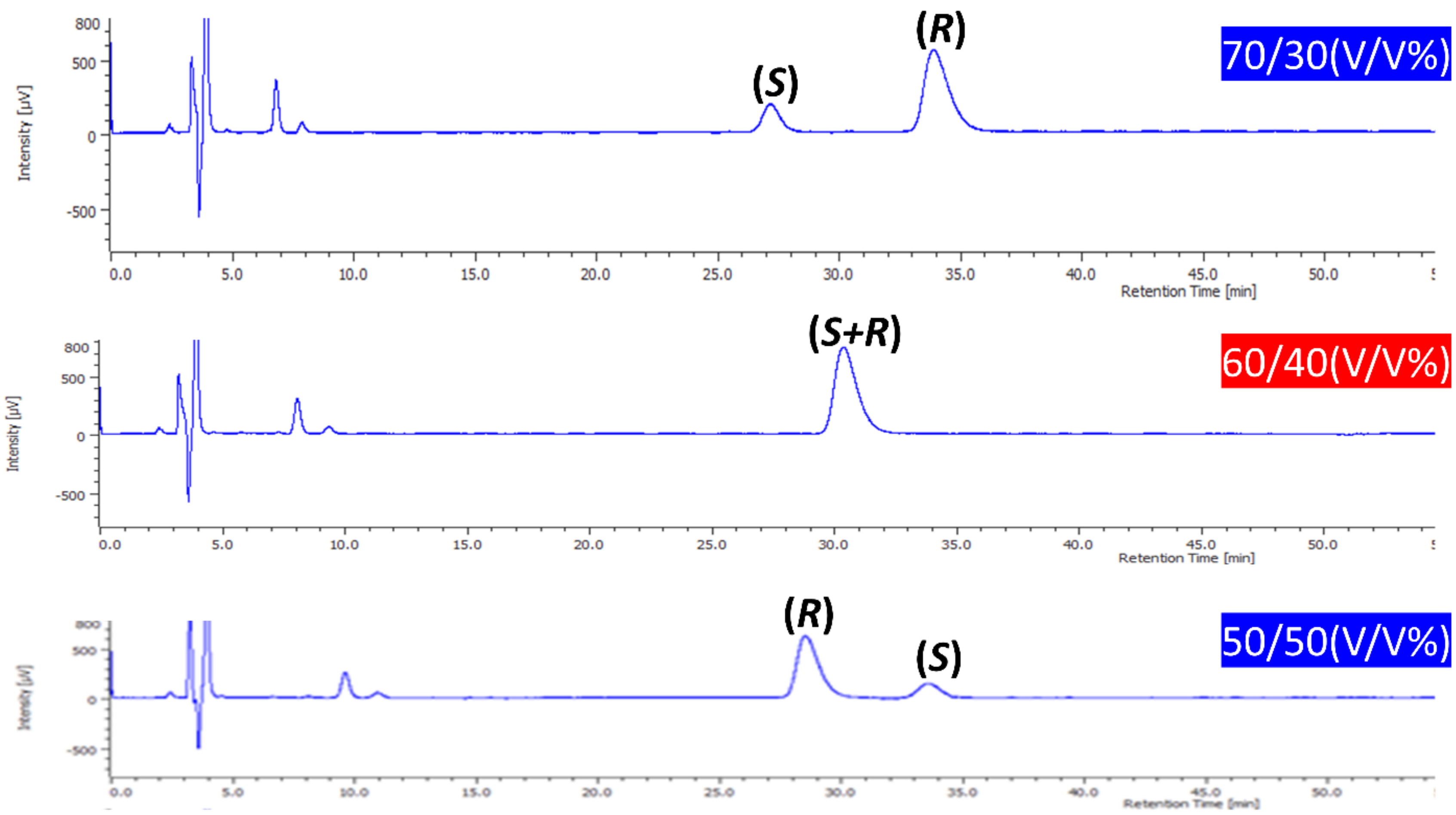

Supplementary Figure S5. Enantiomer elution order reversal of THAL in different MeOH/IPA mixtures on Chiralpak AD column. Flow rate: 0.5 mL/min. Temperature 25 °C.

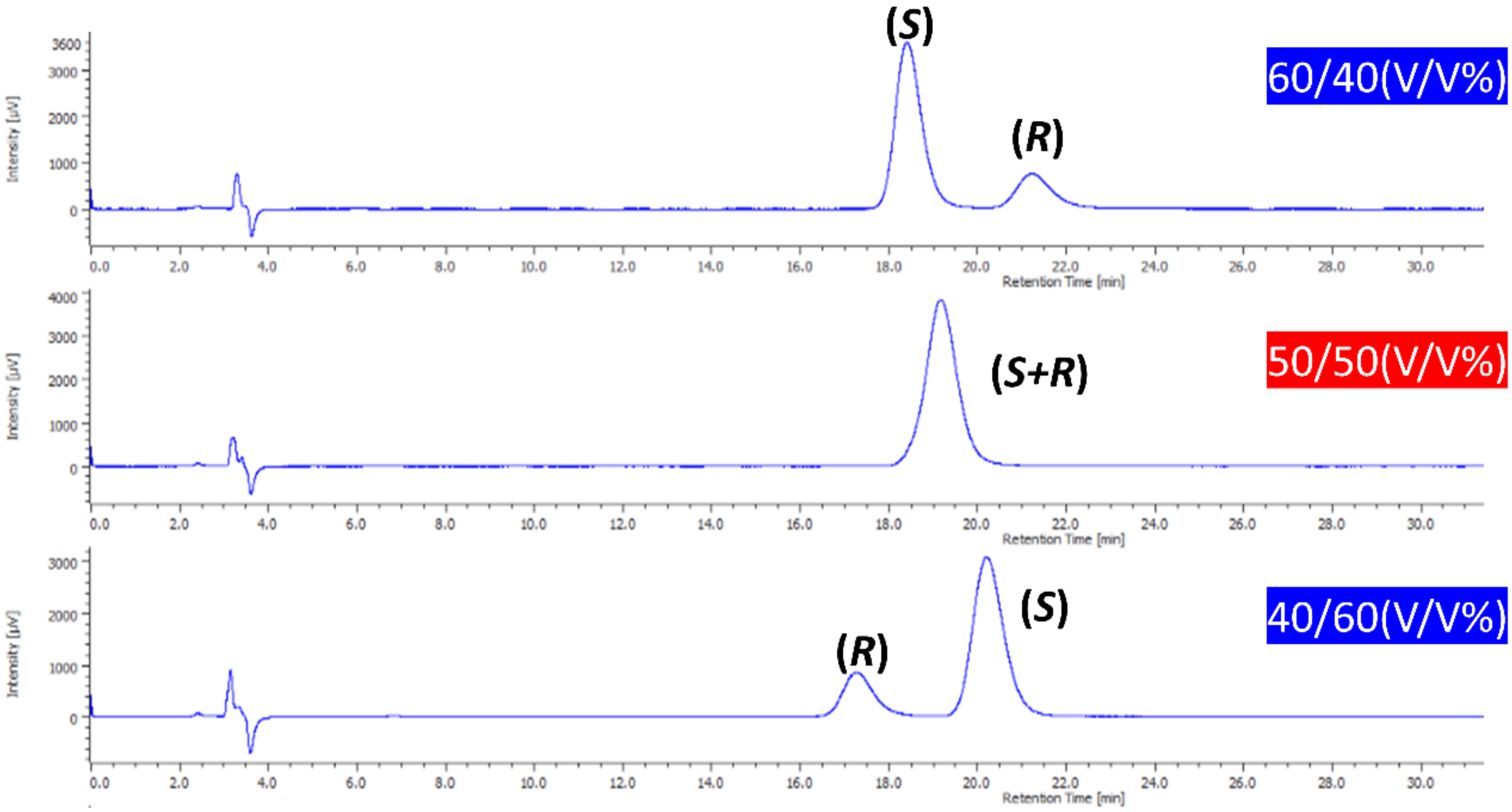

Supplementary Figure S6 Enantiomer order reversal of POM in different MeOH/IPA mixtures on Chiralpak AD column. Flow rate: 0.5 mL/min. Temperature 25 °C.

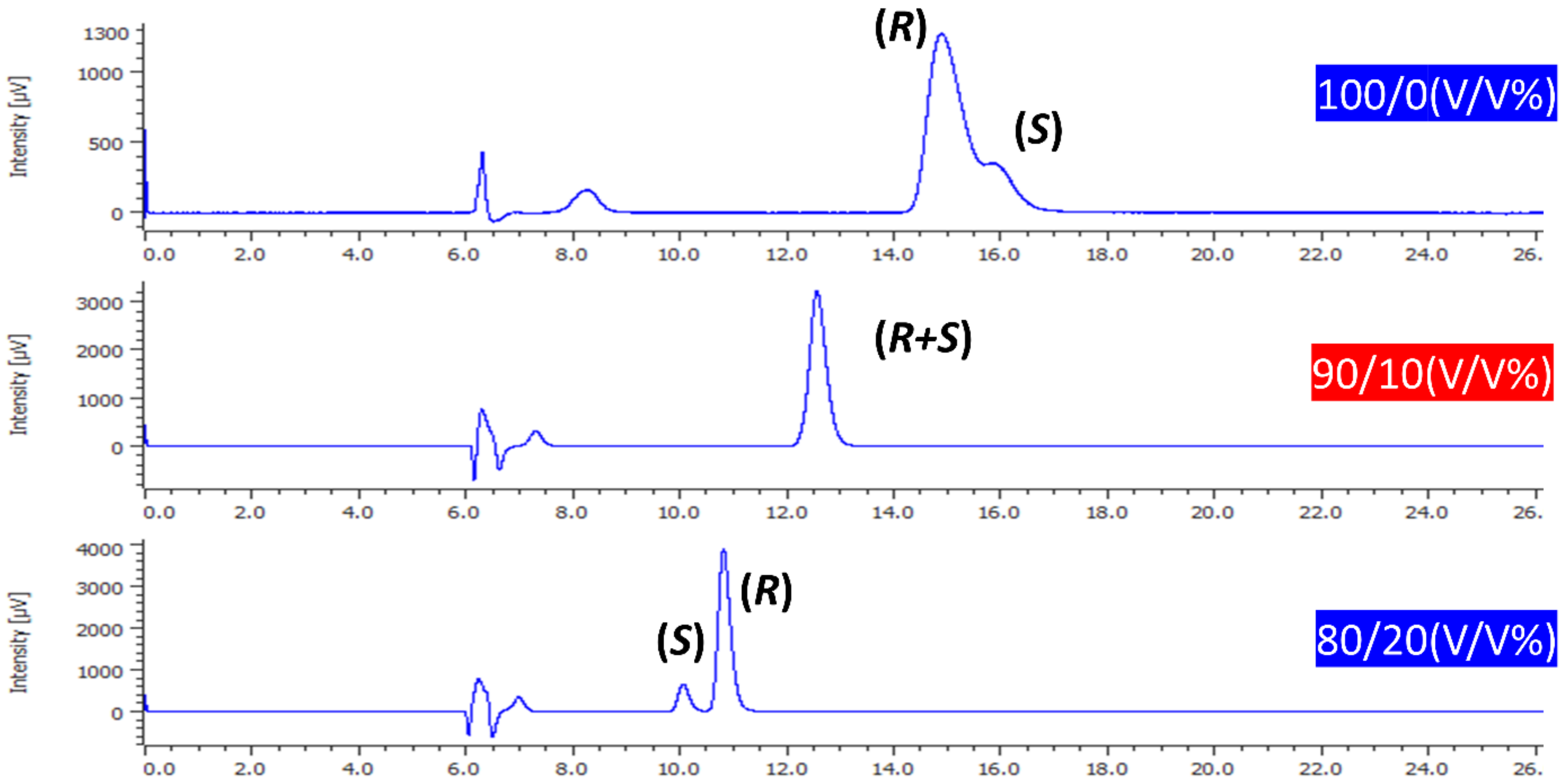

Supplementary Figure S7. Enantiomer order reversal of THAL in different ACN/EtOH mixtures on Chiralpak AD column. Flow rate: 0.5 mL/min. Temperature 25 °C.

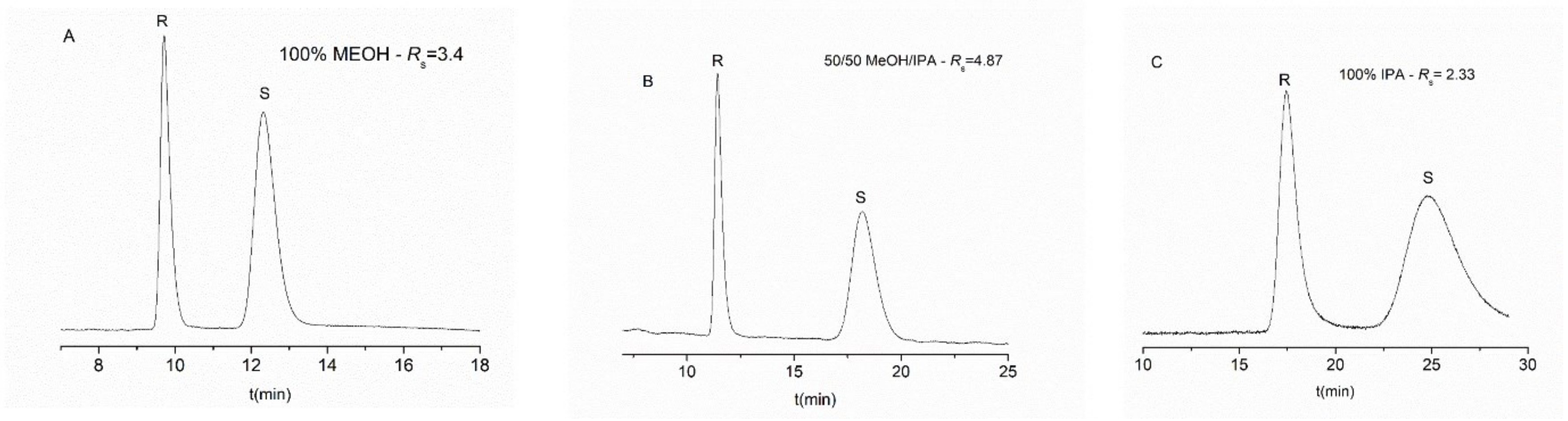

Supplementary Figure S8. POM on Chiralcel OD column using 100% MeOH (**A**), 50/50 MeOH/IPA mixtures (**B**), 100% IPA (**C**) as eluent. The R_s values are 3.40, 4.87 and 2.33, respectively.

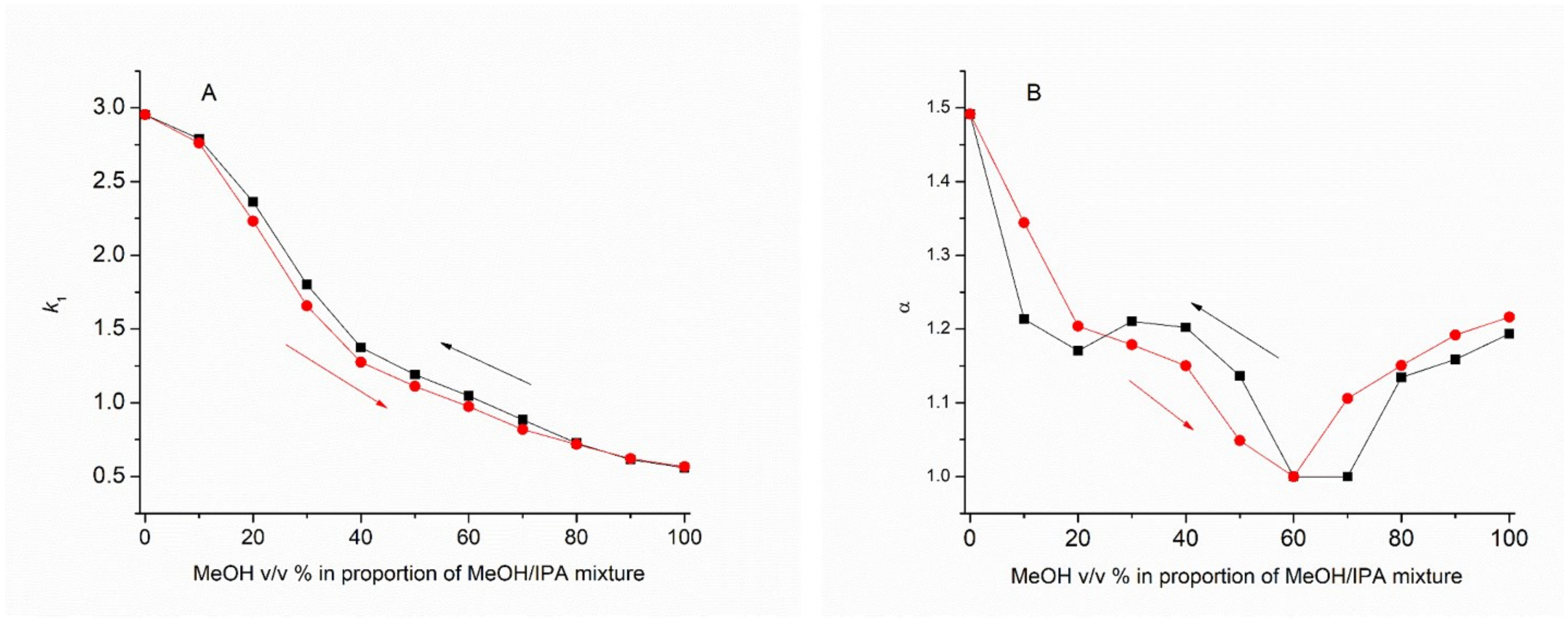

Supplementary Figure S9. Representative graphs of retention factor/separation factor vs. eluent composition A: Retention factor of *R*-POM in different MeOH/IPA compositions on Lux Amylose-2 column, B: Separation factor of POM enantiomers in different MeOH/IPA compositions on Lux Amylose-2 column
